# The Viral Class II Membrane Fusion Machinery: Divergent Evolution from an Ancestral Heterodimer

**DOI:** 10.3390/v13122368

**Published:** 2021-11-26

**Authors:** Pablo Guardado-Calvo, Félix A. Rey

**Affiliations:** Unité de Virologie Structurale, Institut Pasteur, Université de Paris, CNRS UMR 3569, 75015 Paris, France; rey@pasteur.fr

**Keywords:** class II fusion proteins, bunyavirus, alphavirus, structural homology

## Abstract

A key step during the entry of enveloped viruses into cells is the merger of viral and cell lipid bilayers. This process is driven by a dedicated membrane fusion protein (MFP) present at the virion surface, which undergoes a membrane–fusogenic conformational change triggered by interactions with the target cell. Viral MFPs have been extensively studied structurally, and are divided into three classes depending on their three-dimensional fold. Because MFPs of the same class are found in otherwise unrelated viruses, their intra-class structural homology indicates horizontal gene exchange. We focus this review on the class II fusion machinery, which is composed of two glycoproteins that associate as heterodimers. They fold together in the ER of infected cells such that the MFP adopts a conformation primed to react to specific clues only upon contact with a target cell, avoiding premature fusion in the producer cell. We show that, despite having diverged in their 3D fold during evolution much more than the actual MFP, the class II accompanying proteins (AP) also derive from a distant common ancestor, displaying an invariant core formed by a β-ribbon and a C-terminal immunoglobulin-like domain playing different functional roles—heterotypic interactions with the MFP, and homotypic AP/AP contacts to form spikes, respectively. Our analysis shows that class II APs are easily identifiable with modern structural prediction algorithms, providing useful information in devising immunogens for vaccine design.

## 1. Introduction

Many of the human pathogenic viruses are enveloped with a lipid bilayer that must fuse with a cell membrane for infection. The required membrane fusion reaction is catalyzed by a specific viral transmembrane protein, termed “membrane fusion protein” (MFP), which is an important target for the development of antivirals [1] and vaccines [2,3,4]. Given its central role in virus entry, the MFP has been the subject of multiple studies aiming to understand its origin, evolution, and fusion mechanism [5,6,7]. Briefly, the MFP from most viruses belongs to one of three structural classes, I, II, or III. The proteins in the various classes differ in structure, organization at the viral surface, biogenesis, priming mechanisms, and triggering factors, but they all use the same overall mechanism to fuse viral and cell membranes. They are exposed at the virion surface in a metastable conformation, commonly referred to as the prefusion conformation, in which a hydrophobic peptide (or loop) designed to insert into the cell membrane is masked until specific activation. Contacts with the target cell result in lowering of the kinetic barrier maintaining the prefusion conformation, thereby initiating an energetically downhill cascade of events to reach the MFP’s lowest energy folding state or post-fusion form. The fusion peptide/loop inserts into the cell membrane [8] while the protein adopts a transient, extended conformation in which it is at the very opposite end from the viral trans-membrane anchor. Bending in half of the MFP to reach the hairpin conformation characteristic of the post-fusion form brings the segments initially anchored in the two membranes into proximity, forcing membrane apposition. The energy released by this transition distorts both membranes, and overcomes the dehydration force between lipid heads in both bilayers to promote direct contacts between lipid heads, allowing the lipid merger to proceed spontaneously from this point.

Here, we focus on class II MFPs, which are found in enveloped RNA viruses from multiple viral families [9] having unrelated replication machineries, such as positive strand RNA viruses or segmented negative strand RNA viruses. They were also identified in cellular organisms [9,10,11]. The origin and evolutionary history of class II MFPs remain outstanding questions. They were first identified in flaviviruses [12] and alphaviruses [13], and later in several families of bunyaviruses [8,14,15,16] and eukaryotic organisms [9,10,11]. Viral class II MFPs are, in general, about 500 amino acids long, although some have amino-terminal extensions. They feature an elongated ectodomain composed of three β-sheet rich domains, and are anchored in the viral membrane via their C-terminal end. They fold in the ER of the infected cell together with an accompanying protein (AP) within a polyprotein precursor. Upon polyprotein processing by host enzymes (most often a signalase), the two proteins remain together as a heterodimer. The AP acts as a folding chaperone, allowing the MFP to adopt its prefusion conformation, and further regulates its activity to ensure that fusion occurs at the right time and place [17]. The membrane fusion reaction is triggered upon heterodimer dissociation induced by the acidic milieu of an endosome. Once free from the AP, a conformational change of the MFP allows it to transit to its ground-state energy folding state in the form of a very stable trimer of hairpins—the postfusion state—with the fusion loops and the transmembrane regions at the same end of the trimer. With the exception of flaviviruses, in which the MFP forms homodimers with the fusion loop buried at the homodimer interface [12,18,19], in all the other class-II fusion complexes, the fusion loop is buried at the MFP/AP heterodimeric interface in the infectious particle. We term the latter the “canonical” class II viruses, which are the focus of this review. The structurally well-characterized canonical class II virus MFP/AP fusion complexes are: E2/E1 in alphaviruses [18], and Gn/Gc in hantaviruses [16] and in phleboviruses [19]. Amino acid sequence analysis suggests that the Gn/Gc complexes displayed at the surface of nairoviruses, peribunyaviruses, and tospoviruses also correspond to class II fusion machineries [20]. Finally, Rubella virus glycoprotein E1 was also shown to be a class II MFP. This virus also displays two glycoproteins at its surface, E1 and E2, but only the structure of E1 in its postfusion form has been reported [21]. There are not enough orthologs of E2 for AlphaFold to make reliable predictions, and, therefore, the question of whether it belongs to the canonical type of class II AP remains unanswered.

Despite an extensive literature on class-II MFPs, comparatively little is known about their APs, partly because their structures have remained elusive for many years. Here, we review what is known about the structural biology of the class II AP. To provide a broader perspective, we have used AlphaFold, a machine learning approach that has recently emerged as a breakthrough in the prediction of protein structures [22,23]. The AlphaFold software predicts protein structures with atomic accuracy without requiring structural homologs as a template, provided that multiple alignments of enough orthologs are available to evaluate covariance.

We show that, like the MFPs, the class II APs of the *Togaviridae* family, as well as several families of the *Bunyavirales* order of positive-sense and negative-sense single-stranded viruses, respectively, share common structural features: a long β-ribbon immediately followed by an “s-type” domain of the immunoglobulin superfamily (IgSF) [24]. This structural motif has been conserved during evolution despite a much stronger pressure for antigenic drift, or even antigenic shift [25], than that experienced by the actual MFP, which is less exposed on the particles. Overall, our analysis reveals that the accompanying protein in the canonical class II viruses appears to have been present in an ancestral AP/MFP heterodimer that has evolved significantly to constitute the class-II membrane fusion machinery of extant viruses.

## 2. Alphaviruses

Alphaviruses encompass the only genus of the *Togaviridae* family in the recently established *Martellivirales* order of positive-strand RNA viruses [26]. This genus includes the arthropod-borne Sindbis (SINV), Semliki Forest (SFV), chikungunya (CHIKV), and the Venezuelan, Eastern, and Western equine encephalitis (VEEV, EEEV, WEEV) viruses, among others, which cause zoonoses with serious consequences for the human population [27]. Mature virions are about 70 nm in diameter and display a T = 4 icosahedral surface shell formed by 240 copies of a heterodimer composed of glycoproteins E2 and E1. Enclosed within the viral membrane there is an icosahedral nucleocapsid complex containing a single copy of a single-stranded, positive-sense RNA genome encoding for four non-structural and five structural proteins [28]. The proteins that comprise the virion are synthesized as a polyprotein from a subgenomic RNA, and are processed by cellular proteases, producing in the endoplasmic reticulum a p62-E1 heterodimer, which trimerizes to form immature viral spikes that are transported to the cell surface. Furin cleavage of p62 into mature E3-E2 proteins primes the viral spikes in the Golgi apparatus and renders E1 fusion competent. Mature virions bud at the plasma membrane via interactions between the cytosolic tail of E2 and viral nucleocapsids that accumulate in the cytoplasm [29]. It has been shown that all arthritogenic alphaviruses, which are those that cause arthralgia, use an adhesion molecule expressed on epithelial, myeloid, and mesenchymal cells, termed MXRA8, to enter into cells [30]. The structure of the complex shows that the receptor does not bind to any of the subunits separately, but inserts into a canyon between two protomers of the spike. Similarly, LDLRAD3, a low-density lipoprotein receptor type-A module, was shown to act as a receptor for the encephalitogenic VEEV [31]. Structural studies showed that LDLRAD3 wedges into a cleft between two adjacent E2-E1 heterodimers of the VEEV spike, engaging domains A and B of E2 and the fusion loop of E1 [32,33].

**Figure 1:**
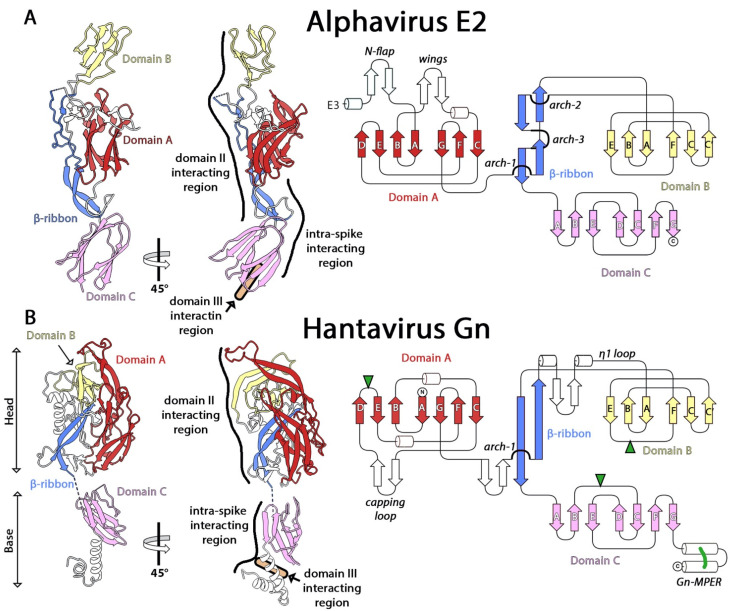
The structure of the accompanying proteins of alphaviruses (E2, panel **A**) and hantaviruses (Gn, panel **B**). The left and central panels show a cartoon representation of E2 (PDB: 6JO8 [30]) and Gn (PDB: 6ZJM [16]) in side view. Domains A, B, C, and the β-ribbon are indicated and colored in red, yellow, pink, and blue, respectively. The right panels show topology diagrams of E2 (top) and Gn (bottom) with the domains colored as mentioned previously.

The structures of the immature p62-E1 heterodimer and mature E3–E2–E1 heterotrimers have been reported [18,34], showing that E2 folds as three distinct Ig-like domains termed A, B, and C, organized around a central “β-ribbon” structure with three characteristic “arches”, which are regions where the chain switches side by arching over the β-ribbon (Figure 1A). Domain A is about 120 residues long and has an Ig-like fold: a β-sandwich with a “c-type” topology, characterized by the presence of β-strand D and the absence of C′ [24]. The Ig-like scaffold contains two insertions, the “N-flap” that contains a protein disulfide isomerase (PDI) motif (C^19^-XX-C^22^), and the “wings”, formed by the BC loop. Domains B and C are also β-sandwiches with an IgSF fold, but of the “s-type” [24], characterized by the presence of β-strand C’, but with the specificity that domain B lacks β-strand G, and has only six β-strands instead of seven as in standard s-type IgSF domains (Figure 1A).

In the mature alphavirus E2/E1 complex, the region of E1 making most of the contacts with E2 is the tip of domain II, including the fusion loop, but also E1 β-strand *b*. The fusion loop is maintained in a groove between domains A and B, and is thus not exposed on infectious particles. In the acidic environment of the endosomes, a region located between the central arch of the β-ribbon and domain B, known as the “acid-sensitive region”, becomes disordered. In turn, domain B moves out and allows exposure of the fusion loop such that it can insert into the cell membrane. In the immature p62/E1 complex, the presence of a linker connecting E3 to domain A clamps the acid-sensitive region in place, and the heterodimer is more resistant to acid treatment. In the spikes, E2 domain C contacts E1 domain III near the viral membrane, and establishes the threefold contacts that stabilize the (E2/E1)_3_ trimer.

## 3. Hantaviruses

Hantaviruses are rodent-borne viruses belonging to the *Orthohantavirus* genus of the *Hantaviridae* family in the *Bunyavirales* order of segmented, negative-strand RNA viruses. This genus includes many important pathogens distributed worldwide. Their transmission to humans can cause two severe diseases: hemorrhagic fever with renal syndrome (HFRS), produced by Old-World hantaviruses, such as Hantaan, Puumala, and Dobrava viruses; and hantavirus cardiopulmonary syndrome (HPS), produced by New-World hantaviruses, such as Andes and Sin Nombre viruses [35]. The virions are pleiomorphic, with some particles roughly spherical at about 120–160 nm in diameter, and others are elongated at about 350 nm in length and 80 nm in diameter [22,36,37]. Gn and Gc form tetrameric spikes that associate laterally to make a square surface lattice. This geometry does not allow the generation of a single closed lattice [16,38], contrary to the icosahedral shell of alphaviruses. The particle encloses the three segments of negative-sense ssRNA that constitute the hantavirus genome, termed small (S), medium (M), and large (L). The genomic RNA is coated with multiple copies of the nucleoprotein (N) that forms a helical nucleocapsid [39] associated with the RNA-dependent RNA polymerase (L protein), forming a ribonucleoprotein complex with each genome segment [38]. The envelope glycoproteins are encoded in the M segment as a precursor polyprotein that is processed in the endoplasmic reticulum by a signal peptidase, producing a Gn/Gc heterodimer that tetramerizes to form the viral spikes [37]. Contrary to alphaviruses, there is no subsequent proteolytic maturation by furin or other proteases, and the priming mechanism for fusion is not understood. Depending on the virus, the virions bud at the plasma membrane or in internal compartments, and are released by exocytosis. Several cell surface molecules have been identified as host factors for entry. They include integrin heterodimers containing subunits β3 and β1 [40,41] for pathogenic and non-pathogenic hantaviruses, respectively; decay-accelerating factor (CD55) [42] for Old-World hantaviruses, and human protocadherin-1 (PCDH1) [43] for New-World hantaviruses. Of all of them, the interaction with PCDH1 is the best characterized, and it has been shown that, similar to alphaviruses, PCDH1 does not recognize the isolated Gn or Gc ectodomains, but binds specifically to intact spikes on the viral surface.

The structure of the N-terminal two-thirds of the Gn ectodomain, known as the Gn “head” (Gn^H^), has been reported both alone [38,44] and in complex with Gc [16]. The C-terminal third, known as the Gn base (Gn^B^), was crystallized as a tetramer [16]. Similar to E2, the Gn ectodomain is folded into three IgSF domains organized around a central β-ribbon (Figure 1B), which also includes one of the characteristic arches observed in alphavirus E2. By analogy with alphaviruses, the domains were termed A, B, and C. Domain A is about 150 residues long and features a “c-type” IgSF fold [24], with one long insertion between β-strands C and D in the form of a β-hairpin that projects the “capping loop” at its distal end, so called as it caps the *cd* fusion loop of Gc in the heterodimer. Domains B and C display an IgSF “s-type” fold [24] with domain B missing β-strand G (as in its counterpart in the alphaviruses). Domain C is followed by a membrane-proximal external region (MPER) formed by an amphipathic 𝛼-helical hairpin stabilized by a conserved disulfide bridge (Figure 1B). Despite the overall similarity between Gn and E2, Gn domains A and B are disposed differently to each other compared with E2, and have developed different insertions into the core domains to interact within the heterodimer.

The conformation of the isolated Gn^H^ is essentially the same as in complex with Gc except for three loops, disordered in the structures of the isolated protein, which are involved in interactions with Gc in the heterodimer. One is the aforementioned capping loop and arch, and the other is the “η1 loop”, which features a strictly conserved stretch of polar amino acids (^281^GEDHD^285^) whose function remains unknown. The C-terminal end of the “η1 loop” makes a parallel β interaction with β-strand *b* of Gc. The interface between the two glycoproteins is further sealed by two conserved glycan chains of Gn, which embrace the tip of domain II. Removal of either of these glycosylation sites has been shown to give rise to defects in intracellular transport of the glycoprotein complex [45]. The fact that the outer lattice lacks icosahedral symmetry and the viral particles are heterogeneous in size has hindered high resolution studies using cryo-EM [38,46], as with the alphaviruses. Instead, a quasi-atomic model of the spike was obtained by combining a low-resolution map obtained by cryo-electron tomography and subtomogram averaging [47] of the surface lattice of Tula virus (a non-human-pathogenic member of the *Orthohantavirus* genus) with the crystal structures of Gn^H^/Gc and Gn^B^ [16] from Andes and Maporal viruses. The resulting model showed that most of the tetrameric contacts are mediated by Gn^B^, which forms an intertwined tetramer composed of domain C and the MPER at the base of the spike.

## 4. Phleboviruses

The *Phenuiviridae* family of arthropod-borne bunyaviruses contains 20 genera, with 137 species inventoried in the latest report of the ICTV [48]. The most relevant phenuiviruses belong to the *Phlebovirus* and *Bandavirus* genera, which include, respectively, the Rift Valley fever virus (RVFV), a mosquito-borne virus that produces acute hepatitis and fetal malformations in several mammalian hosts, and the Dabie virus (DABV, genus *Bandavirus*), which was previously known as the “severe fever with thrombocytopenia virus” (SFTSV), a tick-borne bunyavirus pathogenic for humans. The genome consists of three segments of negative-sense ssRNA, termed S, M, and L based on their electrophoretic mobility. In most species of the family, the M segment encodes two glycoproteins (Gn and Gc) as a precursor polyprotein that is subsequently processed by a signal peptidase in the ER. In RVFV, the M segment contains several translational start codons that are alternatively used to produce a polyprotein precursor to the mature proteins NSm, Gn, and Gc, or p78 and Gc, where p78 encompasses the NSm–Gn fusion, and has a determinant role in virus dissemination within mosquitoes [49]. The Gn/Gc heterodimers produced in the ER transit to the Golgi apparatus (GA), where budding of spherical virions of about 100 nm in diameter [19] displaying a T = 12 icosahedral lattice takes place. Using a genome-wide CRISPR/Cas9 screen, the low-density lipoprotein receptor-related protein 1 (Lrp1) was identified as an essential host factor for entry into cells [50]. These studies also showed a direct interaction between Lrp1 and RVFV Gn.

**Figure 2:**
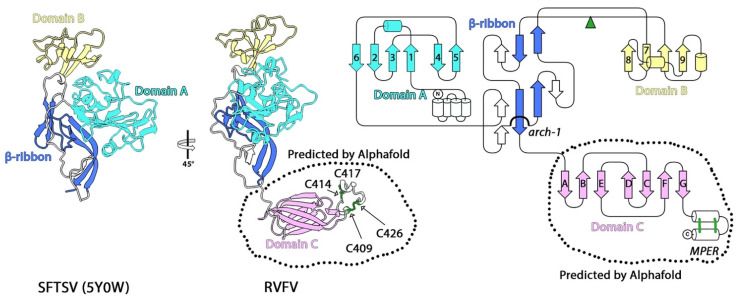
The structure of the accompanying protein Gn of several families of phenuiviruses (phlebovirus and bandavirus). The left panels show a cartoon representation of the models derived from x-ray crystallography from SFTSV (PDB: 5Y0W [53]) in side view. Domains A, B, C, and the β-ribbon are indicated and colored in cyan, yellow, pink, and blue, respectively. The central panels show the structure of the ectodomains of Gn from RVFV obtained using AlphaFold [22]. Regions not present in the experimental crystallographic models are marked with a dashed outline. Disulfide bridges in the MPER region are represented as green sticks. The right panel shows a topology diagram based on the AlphaFold model colored by domains as indicated, the disulfide bonds in the MPER region are shown as green lines, and the location of the putative N-glycosylation site with green arrowheads. The alphafold model of RVFV is available as a Supplementary Items in this manuscript.

The structure of the isolated N-terminal two-thirds of Gn, the Gn “head” (pGn^H^ for “phlebovirus Gn^H^”, so termed to distinguish it from hantavirus Gn^H^, or hGn^H^)), has been reported both for RVFV and DABV [21,51,52], the latter in complex with a neutralizing antibody. Both structures display a similar overall fold with two domains organized around a central β-ribbon, including the characteristic arch observed in hGn^H^. Although the structures of these domains are different from that of their hantavirus counterparts, for the sake of clarity, we also term them A and B (Figure 2). Domain A is a six-stranded β-sandwich with an N-terminal extension formed by a bundle of four α-helices, whereas domain B displays a mixed α/β fold. The remaining C-terminal third of the pGn ectodomain is not present in the reported structures. Size exclusion chromatography, mass spectroscopy, and site-directed mutagenesis experiments performed with the recombinant baculovirus-produced intact ectodomain revealed the presence of disulfide-bonded homodimers involving the last four cysteines of the Gn ectodomain, and identified a disulfide bond formed by cysteines 356 and 424 (DABV numbering) as critical for stabilizing Gn [53].

We used AlphaFold [22] to predict the structure of full length Gn (residues 154–690) of the RVFV polyprotein precursor (Uniprot code: P03518). The program produces a per-residue confidence metric termed pLDTT on a scale from 0 to 100 [22]. pLDTT values higher than 70 reflect reliable models with correct backbone predictions, and those with values higher than 90 correspond to models with both reliable backbone and side-chain orientation predictions. pLDTT values lower than 50 are a strong predictor of disorder, and regions with such values are either unstructured under physiological conditions or only structured as part of a complex. The AlphaFold prediction of the RVFV Gn fold (Figure 2, central panel) has very high confidence with an average pLDTT of 85.6, and revealed that the head domain, which aligns closely with the structure of RVFV pGn^H^ determined by x-ray crystallography (PDB: 5Y0W), is followed by an extended linker of nine amino acids, a β-sandwich with the same “s-type” IgSF fold observed in domain C of alphavirus E2 and hantavirus Gn, followed by a C-terminal, and an amphipathic α-helical hairpin stabilized by two intramolecular disulfide bonds. The structure of the latter segment is very similar to the MPER present on hGn, and we, therefore, term it the phenuivirus Gn MPER (pMPER, Figure 2). As mentioned above, the hantavirus Gn MPER interacts with the viral membrane in the spike, and it is likely that the interactions with a membrane are important for Gn to acquire its correct fold in this region. Thus, overexpression of the soluble ectodomain may lead to pMPER being partially misfolded, and its four cysteines, instead of forming intramolecular disulfide bonds, remain free and generate cross-linked homodimers. The disulfide bond between Cys^356^ and Cys^424^ (Cys^333^ and C^403^ in the RVFV numbering), identified as critical for Gn stability [53], is in the hydrophobic core of domain C. Taken together, the prediction is compatible with the available biochemical data, and suggests that the β-ribbon, domain C, and the helical hairpin in bunyaviruses is the invariant core of the class-II AP fold.

The precise organization and specificity of Gn/Gc spike interactions have remained elusive because of an inherent malleability of the viral particles, which deviate from having perfect icosahedral symmetry. This feature has limited high-resolution cryoEM structural studies. To date, the cryo-EM map with the highest resolution reaches just beyond 8 Å. This map was obtained by applying a localized reconstruction procedure [54] on particles previously fixed with formaldehyde [19]. The models of RVFV Gn^H^ and Gc were docked individually into this map using a molecular dynamic fitting approach [55] and resulted in a quasi-atomic description of a large part of the icosahedral shell. This fitting showed that Gn and Gc assemble as heterodimers such that domain A interacts with the tip of domain II of Gc. Although higher resolution reconstructions would be needed to define the exact nature of these interactions, the resolution is enough to conclude that the fusion loop of Gc is shielded from solvent at the interface between domain A and B, reminiscent of the alphavirus spike organization.

## 5. Tospoviruses

The *Tospoviridae* family of plant bunyaviruses has a single *Orthotospovirus* genus containing 26 species reported by the ICTV [48]. They are transmitted from plant to plant using thrips as vectors, and represent a major agricultural concern, causing numerous economic losses [51]. The prototype of the family is Tomato spotted wilt virus (TSWV). As with the other bunyaviruses mentioned above, the tospovirus genome is made of three segments of ssRNA of negative polarity. The envelope glycoproteins are encoded as a single precursor that is co-translationally processed by a signal peptidase to yield two mature glycoproteins, Gn and Gc, which form a complex in the ER that transits to the GA where newly formed virions bud [52]. To this date, we do not know how both proteins are organized at the surface of the mature virion.

**Figure 3:**
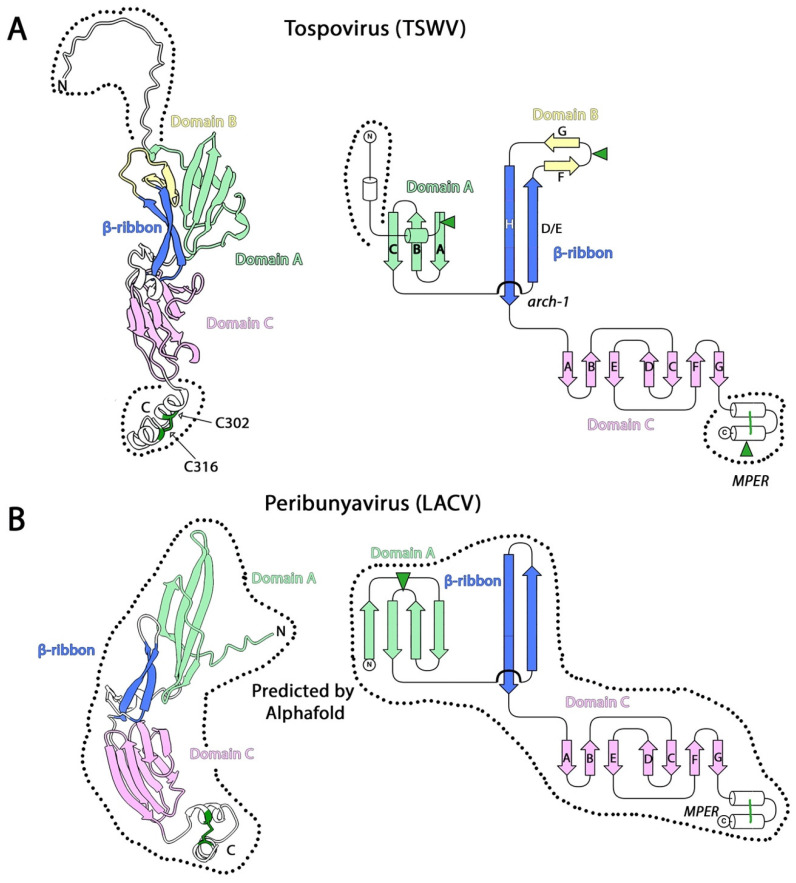
The structure of the tospovirus (**A**) and peribunyavirus (**B**) class II AP. Shown are the AlphaFold prediction of the tospovirus TSWV Gn (R9RUV9, panel **A**) and LACV Gn (Q8JPR1, panel **B**) colored according to domains as indicated. The left panels show a cartoon representation of the model, and the right panels a topology diagram. In A, the dashed outline indicates the regions not present in the X-ray crystallography model of TSWV Gn (PDB: 6YA2 [56]). To date, there is no experimental structure for Gn of any peribunyavirus, and only the prediction by AlphaFold is displayed in B. The disulfide bond in the MPER regions is indicated with green lines, and the location of the putative N-glycosylation site with green arrowheads. These models are available as a Supplementary Items in this manuscript.

The crystal structure of the isolated ectodomain of Gn from TSWV has been reported and shown to comprise three domains sequentially named N-terminal (NTD), pincer (PD), and C-terminal (CTD) domains [56]. The NTD is about 60 residues and comprises an α-helix, a three-stranded, antiparallel β-sheet, and a glycosylation site (Figure 3A). Topologically, the NTD domain would correspond to domain A described in other class II APs, but shows no structural similarity to them. The 80 most *N*-terminal residues of Gn were not resolved in the reported structure. The PD is about 60 residues and comprises a long β-ribbon formed by two contiguous β-strands (D and E) arching over a long β-strand H and running parallel to it. The turn between β-strands E and H contains a two-stranded β-sheet and one glycosylation site. Structurally and topologically, the β-ribbon present in the PD would be equivalent to that previously described in the class II APs of alphaviruses, hantaviruses, and phenuiviruses, and the two-stranded β-sheet inserted in the turn a remnant of domain B [56]. The CTD is about 90 residues and comprises a β-sandwich with an “s-type” IgSF fold [24] stabilized by a single disulfide bond between β-strands A and G, similar to domain C of hantavirus Gn. This globular domain is followed by an α-helix displaying a free cysteine (C^302^ in the TSWV numbering), which is responsible for the disulfide-mediated dimerization observed when the soluble Gn ectodomain is produced. However, 14 residues further down in the sequence, there is another cysteine (C^316^) that was absent in the cloned ectodomain terminating at residue 313, which could potentially form a disulfide bond with C^302^, and would form an α-helical hairpin similar to that observed in hantavirus Gn. To explore this idea, we used AlphaFold to predict the structure of the complete TSWV Gn protein (residues 31–368, Uniprot code: R9RUV9). The model thus generated predicted the formation of a hairpin similar to that reported for hantaviruses, and suggests that the segment (^310^IYKQTACINFS^320^), which the UniProt database partially predicts as part of a TM segment (underlined) and contains an *N*-glycosylation sequon (in bold), is actually the C-terminal helix of the tospovirus Gn MPER (Figure 3A).

## 6. Peribunyaviruses

The *Peribunyaviridae* family of arthropod-borne bunyaviruses features 4 genera and 112 species reported by the ICTV [48]. The members of the *Orthobunyavirus* (OBV) genus are transmitted by infected mosquitoes or midges, and many of them cause severe disease in humans and farm animals. The prototype of this genus is Bunyamwera virus (BUNV), but the most medically relevant species are Oropuche virus (OROV), which causes acute febrile illness in some regions of South America [57], La Crosse virus (LACV), which causes pediatric encephalitis in North America [58], and Schmallenberg virus (SBV) [59], a ruminant pathogen that recently emerged in Europe. As in the other bunyaviruses, the OBV envelope glycoproteins are encoded as a single precursor polyprotein that is co-translationally processed by a signal peptidase to yield two mature glycoproteins, Gn and Gc, and a non-structural protein, denoted Nsm, encoded in between. Unlike the glycoproteins of hantaviruses and phenuiviruses, which are of similar size, in OBVs, Gn is about 200 aa and Gc is about 900 aa long. The second half of Gc is highly conserved across OBVs, and is predicted to display the typical class II MFP fold, whereas its N-terminal half is variable, and was furthermore shown to be dispensable for virus entry into cells [60]. Despite the importance of peribunyaviruses in human and animal health, there is little structural data on the glycoproteins. To date, only two structural studies have been published, a low-resolution cryo-electron tomography (cryo-ET) reconstruction [61] on BUNV particles, showing prominent trimeric spikes and the crystal structure of the N-terminal half of Gc and its docking into the cryo-ET reconstruction [62]. This work showed that the N-terminal half of Gc forms the prominent spikes, and that its C-terminal half in complex with Gn lies flat on the membrane, forming a “floor” connecting three adjacent spikes in the particle.

We have predicted the structure of the full length Gn (residues 14–299) of the LACV polyprotein precursor (Uniprot code: Q8JPR1) using AlphaFold [22], and obtained a reliable model with a pLDTT of 85 (Figure 3B). The model revealed an N-terminal four-stranded β-sheet (residues 14–72) interacting with a long β-ribbon with no trace of domain B. As in the other class II APs described above, the C-terminal domain (residues 109–174) is a β-sandwich with an “s-type” IgSF fold that is followed by an α-helical hairpin stabilized by a disulfide bond. This structural prediction suggests that Gn from OBVs is closely related to that of tospoviruses.

## 7. Nairoviruses

The *Nairoviridae* family of tick-borne bunyaviruses contains 7 genera with 47 species reported to date by the ICTV [48]. Crimean-Congo hemorrhagic fever virus (CCHFV, genus *Orthonairovirus*) is the most relevant nairovirus, and is the prototype of the family. It is transmitted to humans upon a bite by an infected tick, or by contact with blood or tissues of infected livestock. CCHFV is not pathogenic in most vertebrates, but, in humans, it causes hemorrhagic fever with a case fatality rate up to 30% [63]. The genome is made by three segments of negative-sense ssRNA, termed S, M, and L, which code for a nucleoprotein (S segment), an RNA-dependent RNA-polymerase (L segment), and a poly-glycoprotein precursor of the envelope proteins (M segment). The biogenesis of the nairovirus fusion machinery is more complex than that of the bunyaviruses discussed above, and requires several cellular proteases for processing the precursor poly-glycoprotein. In the ER, a signal peptidase generates first a Gn precursor (preGn), a Gc precursor (preGc), and a nonstructural double-membrane spanning protein (NSm). preGn and preGc are transported to the Golgi apparatus where the mucin-like domain (MLD) of preGn is heavily O-glycosylated [64], and both preGn and preGc are cleaved by a SKI-1/S1P serine protease to yield the mature glycoproteins Gn and Gc. This cleavage also releases the soluble N-terminal product of preGn, gp160, which contains the MLD and a 38 kDa protein, termed gp38. gp160 is further cleaved by furin at a well-conserved RSKR motif located at the junction of the MLD and gp38. Experiments with a reverse genetics system showed that the MLD is dispensable for the folding and trafficking of preGn, but the truncation of gp38 causes Gn to be retained in the ER, suggesting that the gp38 has a chaperone activity [65].

**Figure 4:**
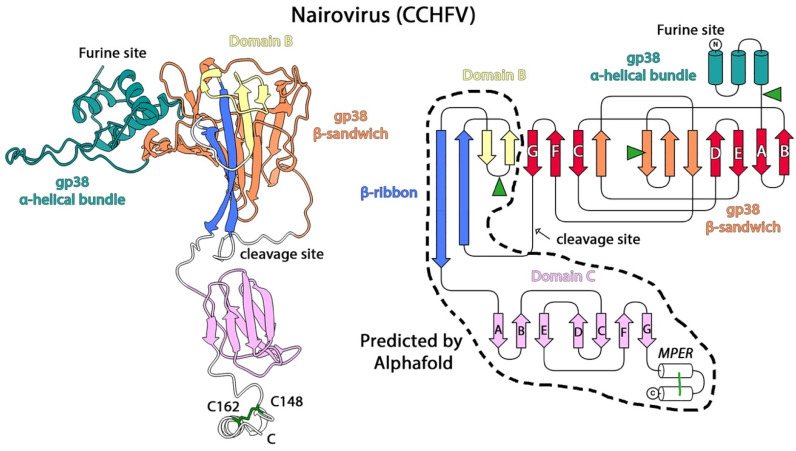
Structure of the nairovirus class II AP. The left panel is a cartoon representation of the CCHFV gp38/Gn complex obtained using AlphaFold. The portion corresponding to gp38 is identical to its x-ray structure (PDB: 6VKF [68]), and is colored according to domains, the N-terminal α-helical bundle in teal, and the C-terminal β-sandwich in orange. The portion corresponding to Gn, for which there is no experimental data, is colored according to domains as indicated. In the right panel, the topology diagram is colored in the same way, and has a dotted outline for the region for which no experimental structure is available. The disulfide bond in the predicted MPER region is depicted and labelled, with the corresponding cysteines labelled in the right panel. As the mucin-like domain (MLD) is intrinsically disordered, it is not included in the diagram. The putative N-glycosylation sites are indicated with green arrowheads, the putative disulfide bond in the MPER (labelled in the right panel) with green sticks (left panel) or a green line (right panel). This model is available as a Supplementary Items in this manuscript.

There are few structural studies on nairoviruses, partly because CCHFV has to be handled in bio-safety level 4 (BSL-4) laboratories. Hazara virus (HAZV), a non-pathogenic nairovirus that belongs to the same serogroup as CCHFV, is used as a model for nairovirus studies [66]. HAZV has been imaged by cryo-ET combined with subtomogram averaging [67], showing spherical particles of relatively uniform size at a diameter of about 100 nm with spike-like projections of 10 nm with tetragonal symmetry, similar to those observed in hantavirus particles. The only glycoprotein for which the structure is known is gp38 [68], although its function remains unclear. It is not highly conserved throughout the family, but gp38 is the target of the monoclonal antibody 13G8, which is non-neutralizing, but was shown to protect against a heterologous CCHFV challenge in a STAT1-knockout mouse model [68]. gp38 folds as an N-terminal 3-helix bundle followed by a β-sandwich composed of a seven- and a four-stranded β-sheet facing each other (Figure 4). The structure is further stabilized by four disulfide bonds, three of them conserved across the family, indicating a similar protein fold. A search of the PDB database for structural homologues showed no matches, but an alignment of the gp38 and Gn sequences of several nairoviruses revealed that some sequence and structural elements were conserved in the region corresponding to the β-sandwich, suggesting that both proteins may have the same fold, and may have arisen through a gene duplication event [68].

We used AlphaFold-2 [22] to predict the structure of the CCHFV poly-glycoprotein segment comprising gp38 and Gn (residues 248-842, Uniprot code: Q8JSZ3), and obtained a reliable model with an average pLDTT of 75 (gp38 has a pLDTT of 81 and the Gn ectodomain 66) (Figure 4). The model revealed that the N-terminus of Gn (residues 520–592 in CCHFV numbering) is folded as a long β-ribbon. Similar to our description above for tospovirus Gn, the turn between strands in the β-ribbon contains a two-stranded β-sheet and one conserved glycosylation site, which would represent a minimal version of domain B, and resembles the glycan reported for tospovirus Gn. In the model for the gp38/Gn complex predicted by AlphaFold, one of the β-strands of domain B interacts with one of the β-sheets of the β-sandwich of gp38, suggesting that gp38 and Gn may form a complex in the mature particle. We therefore propose that gp38 could be the equivalent of domain A as observed in other APs, with the difference that there is a cleavage site in the linker that connects it to the β-ribbon, and so the complex can eventually dissociate upon cleavage. Topologically, the β-sandwich shows the same topology with DEAB and GFC β-sheets composing the β-sandwich of domain A in alphaviruses (compare Figure 1 with Figure 4), except that there are insertions that make additional strands (in orange in Figure 4). In the β-sandwich, the smaller GFC β-sheet faces the orange side of the large β-sheet (Figure 4), which may reflect a very long evolution of this domain from domain A of a potential ancestral precursor class II AP. In CCHFV Gn, the C-terminal domain is again a β-sandwich with an “s-type” IgSF fold [24] followed by an α-helical hairpin stabilized by a disulfide bond, the same organization observed in all other bunyaviruses. Although experimental validation is required, the AlphaFold model suggests that the fold of nairovirus Gn is a minimalist version of the Gn fold reported for other bunyaviruses, and that the gp38-Gn moiety of the precursor may derive from a single class II AP.

## 8. Common Features of the Canonical Viral Class-II AP

**Figure 5:**
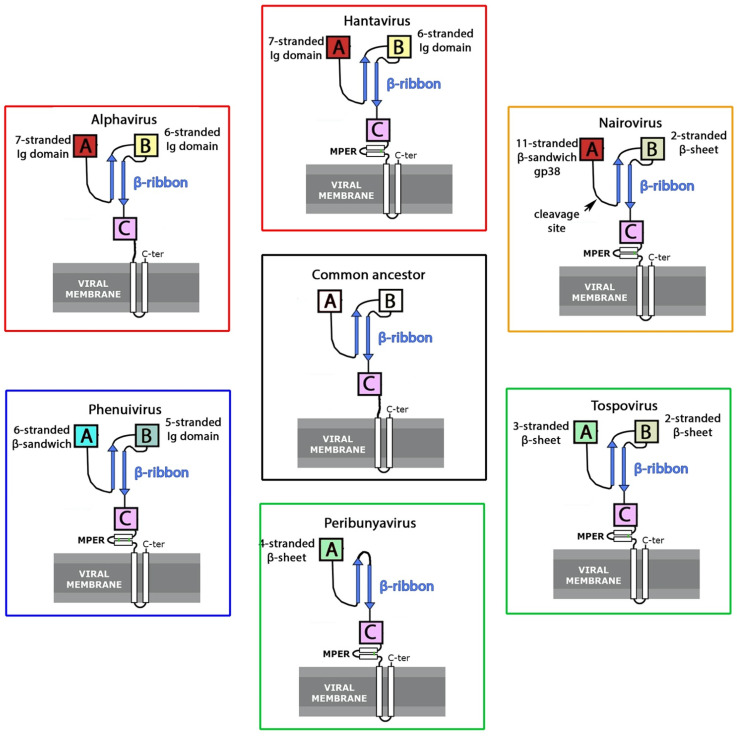
Diagram displaying the relationship between class-II accompanying proteins. Based on structural data, we propose that most class II AP derive from a common ancestor from which they have evolved, although alternative possibilities are plausible (see text). We can classify the class II APs of extant viruses within three groups according to their structural characteristics. The AP of alphaviruses and hantaviruses constitute the first group (indicated by a red square), those of orthobunyaviruses and tospovirus the second one (green square), and the AP of phenuiviruses forming a third group (blue square). The AP of nairoviruses would be somewhere between the first and second group (orange square).

One of the defining characteristics of viral class II MFPs is that to be functional, they must fold as a heterodimer with an accompanying protein, which then multimerizes to create a surface lattice during particle morphogenesis. The resulting complex regulates where and when the MFP is released to undergo its fusogenic conformational change for entry into a target cell. In addition, at least in some alphaviruses and hantaviruses, the AP is directly involved in the recognition of a cellular receptor to induce the required endocytosis step [30,43]. Despite the key role played by APs in the viral cycle, which makes them prime targets for the development of antiviral therapies and vaccines, their structural characterization has not advanced at the same pace as that of their partner, MFP, in the fusion machinery. Here, we have reviewed the common structural features of the class-II Aps of alphaviruses and different families of bunyaviruses, showing that they share a core structural motif: a long β-ribbon decorated with characteristic arches, followed by an “s-type” IgSF domain C, according to the alphavirus nomenclature (Figure 5).

The β-ribbon moiety, together with domains A and B (which are not always present), mediates the heterodimeric interaction with the fusion protein, whereas domain C directs spike formation, essentially, via homo-oligomeric interactions. On top of this conserved motif, there are two accessory domains characteristic of each viral family: an N-terminal domain A and a central domain B at the turn of the β-ribbon. Contrary to domain C, these domains are exposed at the top of the spike, where they are highly exposed to the humoral immune system and display the most variability. Domain A displays a “C-type” Ig-like fold in alphaviruses and hantaviruses, is reduced to a single β-sheet in tospoviruses and OBVs, and, in nairoviruses, is a large and cleavable protein, termed gp38, containing a β-sandwich that partially retains the topology of domain A seen in alphaviruses and hantaviruses. Domain B is a six-stranded β-sandwich with identical topology in alphaviruses and hantaviruses, but is a mixed α/β structure in phenuiviruses, a simple β-hairpin in tospoviruses and nairoviruses, and is altogether absent in OBVs.

The long β-ribbon appears to be used to accompany the long domain II of the MFP, with the fusion loop at the distal end. It has evolved alternative domains A and B to mask the conserved fusion loop while providing the required variability to avoid the antibody response of the vertebrate host. The limited extent of domain B in tospoviruses could be related to the lack of an antibody response in plants and in the arthropod vectors. Likewise, its absence in OBVs, which, like the tospoviruses having a small domain A, is likely related to the large and highly variable N-terminal domain of the MFP of these viruses, which was shown to carry the main antigenic determinants [62].

In addition to the invariant β-ribbon followed by domain C displaying an identical topological arrangement of the β-strands, the bunyavirus class II APs have additional features in common, highlighting a closer evolutionary relationship. Domain C always contains a cysteine residue located at the N-terminal end of β-strand A, which either forms a disulfide bridge with β-strand F (hantaviruses, tospoviruses, peribunyaviruses, and nairoviruses) or with the linker that connects to the β-ribbon (phenuiviruses). Furthermore, a membrane-proximal external region (MPER) in the form of an α-helical hairpin stabilized by one or two disulfide bonds is present between domain C and the transmembrane segments of Gn in all the bunyaviruses for which we examined the structure.

As a whole, the combination of experimental data and AlphaFold predictions point to a common origin for the class II membrane fusion machinery, and not just the MFP. If the homology in the AP has not been evident up to now, it is because the two proteins that make up the complex are not under the same evolutionary pressure, and the Aps have evolved much faster. Our observations point to an ancestral fusion machinery with an AP containing the β-ribbon, domain C, as well as domains A and B, with a topological arrangement of β-strands as observed in the hantaviruses, as suggested by the similarity in their 3D fold observed with the very distant alphaviruses. In this scenario, these domains would have been replaced in the case of the phenuiviruses, nairoviruses, and tospoviruses, and lost during evolution in the case of the OBVs. Alternatively, the original AP could have been the minimal version as seen in OBVs and tospoviruses, and a more recent horizontal exchange event could have taken place from hantaviruses to alphaviruses. It is impossible, with the available data, to provide a timing of events.

## 9. Evolutionary Considerations

Our observations support the notion that the class II membrane fusion machinery has evolved from a common ancestor, despite its presence in viruses belonging to four different orders of RNA viruses according to the current taxonomy (https://talk.ictvonline.org/taxonomy/, accessed on: 20 November 2021): alphaviruses in the *Martellivirales*; Rubella virus in the *Hepevirales*; flaviviruses in the *Amarillovirales*; and hantaviruses, phenuiviruses, nairoviruses, tospoviruses, and peribunyaviruses in the *Bunyavirales*. This classification is informative about the evolutionary pathway of viruses in general. The various orders of RNA viruses classified by the ICTV are based mainly on the polymerase gene. The fact that they all independently acquired ortholog genes for particle assembly and the subsequent cell entry step is a further illustration of the mosaic nature of viral genomes, which are composed of genes of different origins assembled together. This is further stressed by the fact that different members within the above-mentioned orders have unrelated genes for their MFPs (i.e., the Arenaviruses in the *Bunyavirales* order have a class I fusion protein), and some include enveloped viruses, as well as non-enveloped viruses, which have genes for a capsid protein and no MFP (i.e, the hepatitis E virus in the *Hepevirales* order). This observation is in line with the enveloped herpes viruses being related to non-enveloped tailed bacteriophages, which have acquired very different machineries to invade cells, whereas their sets of core genes for their DNA-genome replication and encapsidation are related [69].

The question that remains is how these genes ended up in the genome of unrelated viruses: were they picked independently from a common gene pool, or have different viruses exchanged them horizontally during evolution? In this context, the flaviviruses are an interesting case since, compared to the other class II viruses, they have different requirements to assemble infectious particles. Flavivirus virions bud in the ER of the infected cell, which is at neutral pH, and are then transported through the secretory pathway, where they are exposed to the mildly acidic pH of the trans-Golgi network (TGN). Upon release into the extracellular milieu, they must be able to fuse when up-taken by another cell into an early endosome, where the pH is also mildly acidic. To avoid triggering fusion in the TGN during exit of the producer cell, and still release virions that are fusogenic at mildly acidic pH during entry, flaviviruses require a more complex assembly pathway that involves budding of non-fusogenic immature particles, which are transformed in the TGN via proteolysis by furin. Flaviviruses thus appear to have adapted their fusion machinery to their particular life style, which led, during evolution, to the loss of the canonical protein accompanying the MFP, and its replacement by a protein of yet a different origin. Some bunyaviruses have been reported to also bud in the ER of the infected cell [70], others in the GA [71,72,73], and others, such as New-World hantaviruses [74,75] and also alphaviruses [76], bud directly at the plasma membrane. But for all of these canonical class II viruses, the fusogenic conformational change is triggered at a pH that is significantly more acidic pH than the milieu in which they budded and transited, so that there is no apparent requirement for budding immature particles that are later converted to become fusogenic for entry.

Several class II MFPs have been also found in eukaryotic cells, where they participate in the fusion of somatic cells [77], or in the fusion of gametes during reproduction [78]. Their available X-ray structures correspond only to the trimeric post-fusion form [11,79,80,81]. How they are organized in the pre-fusion form, in the presence of an AP or not, is not known. Recently, however, a potential AP was reported for the class II MFP (termed protein HAP2 or GCS1) of the green alga *Chlamydomonas reinhardtii* [82]. The trans-membrane protein MAR1, which is present at the surface of *Chlamydomonas* gametes, was shown in pull-down experiments to be associated with HAP2 in its pre-fusion form [83]. These authors also showed that in the absence of MAR1, HAP2 mislocalized and did not reach the mating structure where gamete fusion takes place. Such a role in directing the localization of the MFP in cells is one of the hallmarks of the class II AP. Analysis with AlphaFold has indicated, however, that MAR1 is structurally unrelated to the canonical viral class II APs analyzed in this review.

## 10. Conclusions

The lack of sequence similarity and structural information has been an obstacle to establishing parallels between the different families of viruses, to infer functional properties, and, given that in many cases the AP is the main target of neutralizing antibodies [20,84,85,86,87], to develop relevant immunogens for vaccine design. We show here that recent structural prediction algorithms make it possible to unambiguously identify the various modules that make up these proteins, although the exact organization of the canonical MPF/AP complex in each case will require experimental structural studies. Yet, combined with the latest technologies in vaccine development, the structural insights emerging from these analyses have the potential to contribute to the development of novel therapies for some of the highly pathogenic viruses belonging to the viral families described here.

## Supplementary Materials

The following are available online at https://www.mdpi.com/article/10.3390/v13122368/s1. Models of Gn from RVFV (residues 154–690, Uniprot code: P03518), TSWV (residues 31–368, Uniprot code: R9RUV9), LACV (residues 14–299, Uniprot code: Q8JPR1), and CCHFV (residues 248–842, Uniprot code: Q8JSZ3) were generated using a local copy of AlphaFold-2 [22], installed using the open-source code available at https://github.com/deepmind/alphafold (accessed on 1 September 2021). Runs were performed on a Centos 7 workstation with 64 CPUs and 4 GeForce RTX 2080 Ti GPUs, using the casp14 preset and including all PDB templates present in the database. We have deposited the structures as Supplementary Items for this manuscript.
